# Behavioural and psychological symptoms in the older population without dementia - relationship with socio-demographics, health and cognition

**DOI:** 10.1186/1471-2318-10-87

**Published:** 2010-11-30

**Authors:** Rianne van der Linde, Blossom CM Stephan, Fiona E Matthews, Carol Brayne, George M Savva

**Affiliations:** 1 Department of Public Health and Primary Care, University of Cambridge, Institute of Public Health, Robinson Way, Cambridge, CB2 0SR, UK; 2 MRC Biostatistics Unit, Institute of Public Health, Robinson Way, Cambridge, CB2 0SR, UK

## Abstract

**Background:**

Behavioural and psychological symptoms are associated with dementia, but are also present in a significant number of the older population without dementia. Here we explore the distribution of behavioural and psychological symptoms in the population without dementia, and their relationship with domains and severity of health and cognitive impairment.

**Methods:**

The Medical Research Council Cognitive Function and Ageing Study is a two-phase longitudinal study of ageing representative of the population aged 65 and over of England and Wales. A subsample of 1781 participants without a study diagnosis of dementia was included in this study. Information on symptoms including depression, apathy, anxiety, feelings of persecution, hallucination, agitated behaviour, elation, irritability, sleep problems, wandering, confabulation and misidentification, cognitive function, health related factors and socio-demographic information was extracted from interviews with participants and knowledgeable informants. Participants were classified according to the Mini-Mental State Examination and by criteria for subtypes of mild cognitive impairment (MCI). The prevalence of behavioural and psychological symptoms and associations with cognitive function, health and socio-demographics was examined. Co-occurrence of symptoms was tested using factor analysis.

**Results:**

Most symptoms were reported more frequently in those with more severe cognitive impairment. Subjective memory complaints were the strongest independent predictor of reported symptoms, and most were reported more often in those classified as having MCI than in those with cognitive impairments that did not meet the MCI criteria. The pattern of co-occurrence of symptoms is similar to that seen in dementia.

**Conclusions:**

Our results highlight that behavioural and psychological symptoms are prevalent in the cognitively impaired older population, and partly explain the variation observed in previous cohorts of individuals with MCI. Behavioural and psychological symptoms offer a target for intervention and so are an important consideration in the assessment of cognitively impaired older people.

## Background

Behavioural and psychological symptoms are common in dementia. These symptoms include depression and anxiety, psychotic symptoms, wandering, agitated behaviour and sleep disorders, and are collectively known as the behavioural and psychological symptoms of dementia (BPSD). These symptoms confer a large proportion of the social burden of dementia [1,2], and are important targets for intervention in dementia patients [3]. Yet these symptoms are not restricted to those with dementia. Many behavioural and psychological problems are also present in a significant proportion of the non-demented older population [4,5]. Behavioural and psychological symptoms have been associated with cognitive impairments not sufficient for a diagnosis of dementia, although their prevalence and relationship to cognitive impairments are not clear. Two recent reviews have highlighted the variation in estimates of the prevalence of behavioural and psychological symptoms in those with mild cognitive impairment (MCI) and suggested that differences in the settings of studies, the characteristics of participant groups, and the definitions of symptoms and of MCI contribute to this variation [6,7]. We have previously compared the prevalence of BPSD in the older population with and without dementia [4]. In this article we use data from the population-representative Medical Research Council Cognitive Function and Ageing Study (MRC CFAS) to explore the relationship between behavioural and psychological symptoms with the socio-demographic, health and cognitive factors that are used to define different classifications of MCI.

## Methods

### Study participants

The Medical Research Council Cognitive Function and Ageing Study (MRC CFAS), is a multi-centre longitudinal study that is fully described elsewhere [8]. Between 1990 and 1991 a random sample of individuals aged 65 years and older living in five centres representative of rural and urban areas in England and Wales (Cambridgeshire, Gwynedd, Newcastle, Nottingham and Oxford) were contacted, with a response rate of 82%. At baseline, 13,004 participants completed the screening interview. A subgroup of those screened including all participants with severe cognitive impairments and a stratified subsample of the remainder (n = 2640) also completed a more detailed assessment. This included selected items from the Geriatric Mental State Automated Geriatric Examination for Computer Assisted Taxonomy (GMS-AGECAT), Mini Mental State Examination (MMSE) [9] and the multi-domain Cambridge Cognitive Examination (CAMCOG) [10].

Persons diagnosed with dementia (defined as an AGECAT organicity rating case level of O3 or above, n = 587 [11]), an unknown dementia status (n = 3), a MMSE score that is missing or below 18 (n = 243), or Parkinson's disease (n = 26) were excluded. Therefore of the 2640 who undertook the assessment interview 1781 persons were included in this study. The aim of this study was to analyse the psychiatric correlates of cognitive impairment, and so those with underlying psychiatric conditions were not excluded from the study.

### Informed Consent and Confidentiality

MRC CFAS has Multi-centre Research Ethics Committee's approval and ethical approval from the relevant local research ethics committees. All participants gave informed consent and patient confidentiality was not breached.

### Assessment of behavioural and psychological symptoms

Symptoms assessed during the screen, assessment and informant interviews include depression, apathy, anxiety, feelings of persecution, hallucination, agitated behaviour, elation, irritability, sleep problems, wandering, confabulation and misidentification. Symptoms were assessed using interviews conducted with both study participants and their informants, combined with observations made by the interviewer during the course of the interviews. Depression was measured with the Cambridge Mental Disorders of the Elderly Examination (CAMDEX) [12]. Details of the questions used to assess each symptom have been described previously [4].

### Assessment of cognitive function and definition for 'mild cognitive impairment' criteria

Cognitive function was assessed using the MMSE [9] and by classification based on definitions of mild cognitive impairment (MCI) and its subtypes [13]. Using the MMSE, cognitive function was classified as low (18-21), intermediate (22-25) or high (26-30). Classifications of MCI aim to identify individuals with cognitive impairments likely to indicate incipient dementia. Three subtypes are considered, amnestic (A-MCI) including deficits in memory, non-amnestic (N-MCI) in which cognitive impairment is restricted to non-memory domains and multiple (M-MCI) in which both memory and non-memory domains of cognition are impaired [13].

A clinical diagnosis of MCI requires a subjective complaint of memory impairment by either the respondent themselves or an informant. Performance on cognitive tests must fall below a specific threshold, typically below 1 or 1.5 standard deviations of the mean age-specific performance, or if test scores are highly skewed below the 16^th ^or 7^th ^percentiles. To exclude those with possible dementia, general cognitive function must not be severely impaired. Exclusion criteria can include any health or psychiatric problems that might be the alternative cause of cognitive impairment, or a severe impairment in activities of daily living. The operationalisation of these criteria, including the level of subjective memory complaint, the specific cognitive tests and exact exclusion criteria, are not specified.

For the present study, 'subjective memory complaint' was defined as a report of memory problems by either the respondent or an informant at either the screening or the assessment interview. 'Objective memory impairment' was defined using as a score below the 16^th ^age-specific percentile on one or more of the remote memory, recent memory or learning memory domains of the CAMCOG. 'Non-memory impairment' was defined using as a score of below the 16^th ^age-specific percentile on any of the other cognitive domains measured by CAMCOG which includes orientation, language, attention/calculation, praxis, abstract thinking or perception. CAMCOG cut-off scores were age adjusted using five 5-year age groups including 65-69, 70-74, 75-79, 80-84 and 85+ years. General cognitive function was deemed to be severely impaired when participants scored less than 22 on MMSE. No health-related or functional exclusions were applied, since the relationships between these and behavioural and psychological symptoms were of interest in the present study.

MCI subtypes were assigned as follows: All MCI subtypes required subjective memory complaint and no severe impairment of general cognitive function (ie MMSE > = 22). Those with memory impairment but no non-memory impairment were classified as A-MCI. Those with non-memory impairment but no memory impairment were classified as N-MCI. Those with impairments in memory and non-memory domains were classified as M-MCI. Participants were not excluded on the basis of medical or psychiatric problems. Full details of the mapping of MCI in MRC CFAS have been published elsewhere [14].

A 'not impaired' group was defined using the following criteria: normal general cognitive function, no severe functional impairment, normal memory and non-memory test performance. Participants who could not be classified as "not impaired" or MCI using any definition were classified as "other cognitive impairment, no dementia" (OCIND). This heterogeneous group includes both respondents with an objective memory impairment but no subjective memory complaint, and those with impaired general cognitive function but not diagnosed with dementia.

### Covariates

As well as those factors that contribute to the definition of MCI, other factors considered in our analysis included sex, age, institutionalization, vascular co-morbidity, education, and social class. Age was reported continuously as well as being dichotomized into 65-74 years versus 75 years and older. Vascular co-morbidity was defined using self or informant reported history of a heart attack, stroke or diabetes. Institutionalisation was divided into two groups including, independent (living alone or in a warden controlled flat) or dependent (living in a residential home, nursing home or hospital). Level of education was measured in number of years of education and was divided into two groups including less than 10 years and 10 or more years of education. Functional impairment was defined as requiring help at least several times per week with activities of daily living (ADLs) such as washing, cooking for themselves, dressing, or if the respondent was housebound. Data were missing in fewer than 2% of individuals for each covariate and was assumed to be missing at random.

### Statistical analysis

All analyses were performed using Stata 10.0. The prevalence of each behavioural and psychiatric symptom was estimated in cognitive groups. Participants were grouped both by MMSE scores, and by MCI classification. Three MMSE categories were defined including low (18-21), mild (22-25) and high (26-30). MCI classifications were 'not cognitively impaired', 'MCI' and 'OCIND'. Prevalences were also compared across MCI subgroups A-MCI, N-MCI and M-MCI as previously described. Differences in prevalence across groups were tested using likelihood ratio tests. Post-hoc pairwise tests comparing subgroups were not conducted.

Participants were back-weighted in prevalence estimation to adjust for over-sampling in the study population of individuals aged 75 years or older and the subsequent stratified sampling for the assessment interview. The relation between symptoms, subjective memory complaint, objective memory impairment, vascular disease, disability and socio-demographic factors was investigated using both univariate and multivariate logistic regression.

Co-occurrence between symptoms was measured using odds ratios estimated using logistic regression. Univariate analyses and multivariate analyses adjusting for the presence of all other symptoms, MMSE, age and sex were conducted. Factor analysis was used to determine the structure of the co-occurrence of symptoms. The Spearman's correlation matrix relating the symptoms was estimated and this was subjected to the principle factor analysis algorithm. The resulting factor solution was rotated for ease of interpretation using the Varimax rotation. This factor analysis was repeated within each of the MMSE and MCI subgroups described above.

## Results

### Characteristics of study participants

Table 1 shows the characteristics of the study participants stratified by MCI classification. The age of all three groups was similar, but women were more likely to be cognitively impaired. Few individuals were living in institutions. Those without cognitive impairments were substantially more likely to be better educated and in a higher social class, and to have better self rated health. The proportion of participants with reported vascular disease was similar across groups. The OCIND group had lower cognitive function than the MCI group or the NCI group, and a lower prevalence of impairments in ADLs. The OCIND group reported most frequent use of anti-psychotic medication, while the MCI group had greater reported use of anti-depressants.

**Table 1:** Characteristics of the population by MCI classification

	No Cognitive Impairment	MCI	OCIND
Age mean(se)	73.5 (0.28)	73.8 (0.43)	73.5 (0.31)
MMSE mean(se)	27.4 (0.08)	25.4 (0.14)	24.4 (0.18)
Female N(%)	389 (58)	242 (62)	439 (65)
Living in institutions N(%)	8 (1.2)	11 (2.8)	23 (3.4)
Vascular disease N(%)	134 (20)	111 (29)	164 (24)
Taking anti-depressant medication	20 (3.0)	23 (5.9)	29 (4.3)
Taking anti-psychotic medication	2 (0.3)	1 (0.3)	5 (0.7)
Impaired activities of daily living N(%)	66 (10)	69 (18)	140 (21)
More than 9 years education N(%)	310 (46)	108 (28)	153 (23)
Regular contact with family N(%)	374 (73)	222 (83)	397 (79)
Poor self-rated health N(%)	178 (27)	168 (44)	256 (38)
Lower social class N(%)	365 (54)	278 (71)	493 (73)

### Prevalence of symptoms across MMSE cognitive classifications

The prevalence of each behavioural and psychological symptom by MMSE group and MCI classification is shown in table 2. The previously reported prevalence in those with dementia is also shown for comparison. The prevalence of many symptoms was higher in those with worse cognition.

**Table 2:** Prevalence of symptoms across cognitive groups.

	MMSE 18-21 (N = 316)	MMSE 22-25 (N = 659)	MMSE 26-30 (N = 806)	p-value	All without dementia or severe cognitive impairment (N = 1781)
Depression	58 (17.6)	67 (8.3)	73 (7.4)	0.001	198 (8.4)
Apathy	78 (25.2)	98 (13.5)	88 (9.6)	0.000	264 (11.8)
Anxiety	22 (7.2)	29 (4.0)	56 (6.7)	0.147	107 (6.0)
Persecution	44 (12.7)	76 (9.5)	61 (6.8)	0.012	181 (8.0)
Hallucinations	24 (6.7)	27 (3.0)	29 (3.4)	0.046	80 (3.5)
Agitation	23 (7.5)	28 (3.6)	28 (2.9)	0.013	79 (3.4)
Elation	18 (4.7)	20 (2.8)	29 (3.1)	0.252	67 (3.2)
Irritability	61 (17.4)	107 (14.6)	93 (10.8)	0.021	261 (12.3)
Sleep problems	131 (40.3)	302 (45.4)	360 (42.4)	0.366	793 (43.0)
Wandering	3 (0.6)	5 (0.4)	3 (0.2)	0.817	11 (0.3)
Confabulation	2 (0.6)	3 (0.2)	2 (0.1)	0.734	7 (0.2)
Misidentification	18 (6.5)	20 (2.9)	19 (2.8)	0.219	57 (3.1)

	**No Cognitive Impairment** **(N = 670)**	**All MCI** **(N = 389)**	**OCIND** **(N = 676)**	**p-value**	**Dementia** **(Savva et al 2009**[4]**)**

Depression	48 (6.0)	61 (14.5)	79 (8.5)	0.002	20.5
Apathy	67 (9.0)	79 (17.8)	110 (13.1)	0.004	50.3
Anxiety	35 (5.4)	33 (8.0)	36 (5.9)	0.427	8.9
Persecution	41 (5.5)	61 (13.5)	76 (9.3)	0.001	25.4
Hallucinations	17 (2.3)	25 (5.2)	34 (4.3)	0.148	15.1
Agitation	20 (2.6)	20 (5.2)	37 (3.9)	0.228	9.0
Elation	21 (2.8)	17 (5.0)	27 (2.6)	0.221	9.5
Irritability	73 (10.5)	82 (17.4)	100 (12.6)	0.041	28.8
Sleep problems	297 (43.0)	198 (49.0)	276 (38.6)	0.036	42.0
Wandering	2 (0.3)	4 (0.4)	3 (0.2)	0.665	12.8
Confabulation	2 (0.1)	2 (0.2)	3 (0.3)	0.483	5.8
Misidentification	22 (3.5)	12 (2.4)	21 (2.4)	0.524	20.3

	**Amnestic MCI** **(N = 75)**	**Non-amnestic MCI** **(N = 185)**	**Multiple MCI** **(N = 129)**	**p-value**	

Depression	7 (9.3)	29 (14.8)	25 (18.7)	0.586	
Apathy	11 (10.8)	34 (17.9)	34 (23.8)	0.202	
Anxiety	7 (12.2)	12 (5.6)	14 (9.2)	0.374	
Persecution	10 (12.7)	25 (12.3)	26 (16.9)	0.723	
Hallucinations	5 (7.6)	10 (2.4)	10 (8.8)	0.042	
Agitation	1 (2.9)	10 (5.5)	9 (6.4)	0.807	
Elation	5 (12.7)	7 (3.0)	5 (2.2)	0.011	
Irritability	20 (24.7)	35 (15.8)	27 (14.0)	0.240	
Sleep problems	31 (44.1)	97 (48.5)	70 (54.3)	0.649	
Wandering	1 (0.4)	1 (0.2)	2 (0.8)	0.682	
Confabulation	1 (0.7)	0 (0.0)	1 (0.2)	0.603	
Misidentification	3 (3.6)	6 (1.5)	3 (3.4)	0.496	

Prevalence of symptoms N(%) across MMSE groups (top) MCI classification (middle) and MCI subtypes (bottom). All percentages are back-weighted to the population. P-values correspond to a likelihood ratio test of differences across groups.

There was a statistically significant trend to increased depression, apathy, psychosis, agitation, and irritability in those with worse cognition. Inappropriate elation, wandering and confabulation were rare in this sample making differences between groups difficult to detect, nevertheless there is some evidence that each of these is more common in those with worse cognition. Sleep problems were common and reported with equal frequency across MMSE groups. There was no significant difference in the prevalence of anxiety across cognitive groups.

### Prevalence across MCI classifications

Significant differences in the prevalence of depression, apathy, persecution and irritability were seen across MCI groups, with the prevalence of each of these lowest in those without cognitive impairment. Sleep problems were reported least often in the OCIND group (38.6%), and most often in the MCI group (49%). Wandering and confabulation were rarely reported in this sample with little evidence of a difference across groups. Hallucinations, agitated behaviour and anxiety were seen least often in those with no impairment and most often in MCI, but these differences were not statistically significant. There was little difference in the prevalence of misidentification across groups.

Cognitive function was higher in the A-MCI group (mean MMSE = 26.3) than in the N-MCI (mean MMSE = 25.3) and M-MCI (mean MMSE = 24.5) groups (F(2,387) = 10.91:p < 0.001). The only statistically significant differences in symptom prevalence observed across MCI subgroups were a high prevalence of elation in A-MCI and a higher prevalence of hallucinations in those with memory impairments (ie M-MCI and A-MCI). There is some evidence that depression and apathy are more common in those with non-memory impairments (N-MCI and M-MCI) with anxiety more common in those with memory impairments. Feelings of persecution and sleep problems were equally common across all groups.

### Effects of health-related, sociodemographic and cognitive characteristics on presence of symptoms

In univariate analyses (additional file 1: table S1) memory impairment, subjective memory complaint, ADL impairment, and lower MMSE score were associated with higher prevalence of many symptoms. Vascular comorbidity is associated with an increased risk of symptoms of mood including depression, anxiety, apathy and irritability.

After adjustment for all other risk factors (additional file 2: table S2) the effect of objective memory impairment and of MMSE score is largely attenuated, while the presence of subjective memory complaint remains associated with the presence of many symptoms. The association between vascular disease with depression, apathy, anxiety and irritability remains although strength is reduced.

Many symptoms appear less common in older people in multivariate analyses, suggesting younger people with lower cognition were more at risk of BPSD than older people. Depression and sleep problems are more frequently reported in women, while apathy is more common in men. There is a non-significant increase in other symptoms in women, which remains in multivariate analyses.

### Co-occurrence of symptoms

Table S3 in additional file 3 shows univariate and multivariate pairwise associations between symptoms. There are many significant associations between pairs of symptoms which remain after adjusting for the presence of all other symptoms, MMSE, age and sex. Factor analysis (table 3) suggests three factors arising from this observed co-occurrence. The three factor solution included: (a) symptoms of hallucination and misidentification, (b) symptoms of anxiety, apathy and depression and (c) irritability, persecution and wandering. The fourth strongest factor does not include any symptoms with loading greater than 0.3. However the uniqueness of each symptom was high, suggesting that much of the variability of each symptom cannot be explained by this factor model, and there are many statistically significant pairwise associations between symptoms that the factor model does not include (supp table 3). The factor structure is largely consistent across cognitive and MCI groups (table 4) and is similar although with weaker associations to that seen in the population with dementia [4].

**Table 3:** Factor analysis of association between behavioural and psychological symptoms in the population without dementia.

Symptom	Factor1	Factor2	Factor 3	Factor 4	Uniqueness
Agitation	-0.03	0.06	-0.02	0.16	0.96
Anxiety	0.11	**0.40**	0.01	-0.02	0.83
Apathy	0.14	**0.33**	0.18	0.02	0.84
Confabulation	-0.04	0.10	0.02	-0.05	0.97
Depression	0.22	**0.42**	0.11	0.02	0.76
Elation	0.17	0.00	0.22	0.21	0.88
Hallucinations	**0.53**	0.08	0.11	0.03	0.70
Irritability	0.08	0.18	**0.39**	-0.01	0.81
Misidentification	**0.49**	0.12	0.00	-0.01	0.74
Persecution	0.07	0.09	**0.42**	0.01	0.81
Sleep problems	-0.01	0.17	0.11	0.09	0.95
Wandering	0.18	-0.06	**0.32**	0.11	0.85

Factor loadings > 0.3 are shown in bold.

**Table 4:** Factor structure representing the relationships between behavioural and psychological symptoms by cognitive classification.

*MMSE groups*	**Factor 1**	**Factor 2**	**Factor 3**	**Factor 4**
MMSE 18-21	mis/hal/wan/ela	apa/mis/anx/dep	per/irr	
MMSE 22-25	mis/hal	apa/anx/dep	per/irr	
MMSE 26-30	mis/hal	anx/dep/(apa)	per/wan/irr	
*MCI classification*				
Normal	mis/hal	anx/dep	per/wan/irr	
MCI	mis/hal	apa/anx/dep/sle	per/wan/ela/irr	
OCIND	mis/hal	apa/anx/dep	per/irr	wan/(ela)
*MCI Subtypes*				
A-MCI	mis/hal	apa/anx/dep/sle	per/wan/ela/irr	agi/irr
N-MCI	mis/hal/anx/dep	apa/sle	per/irr	ela/agi
M-MCI	mis/hal/wan/ela	apa/anx/dep	irr/(per)	
Dementia (adapted from Savva et al 2009 [4])	mis/hal/con/apa/per/wan	anx/dep/hal	irr/per/agi	agi/ela/hal/sle/wan

Symptoms are included in each factor if their factor loading is >0.3 (symptoms loading >0.25 but <0.3 are shown in brackets). Key to symptoms: mis = misidentification, hal = hallucinations, wan = wandering, ela = elation, apa = apathy, anx = anxiety, dep = depression, per = feeling of persecution, irr = irritability, agi = agitated behaviour, sle = sleep problems.

## Discussion

### Summary of findings

In this large, representative study of the population without dementia or severe cognitive impairment we have reported the prevalence of behavioural and psychological symptoms, and the relationship between such symptoms and aspects of cognitive impairment, adjusting for a wide range of sociodemographic and health related risk factors. The prevalence of many symptoms increases with worsening cognition, the presence of a subjective memory complaint and impaired functional ability. Depression, anxiety and apathy were more common in those with a history of vascular disease whereas agitated behaviour is more common in those without.

Many symptoms were reported more frequently by those with MCI than by those who were cognitively impaired without meeting the definition of MCI (the OCIND group), the majority of who failed to meet the criteria for MCI owing to the absence of a subjective memory complaint. Within the MCI group, those without memory impairments (N-MCI) typically suffered fewer symptoms than those with only memory (A-MCI) or with memory and non-memory cognitive impairment (M-MCI). However, in multivariate analysis adjusting for subjective memory complaint and cognitive function, those with an objective memory disorder were not found to be at higher risk. Age and sex also affected the prevalence of psychiatric conditions, with depression more common in women and in those under 75 years of age, while apathy was more frequently reported in men, echoing our earlier findings in the population with dementia [4]

### Strengths and limitations of the study

We assessed the prevalence of behavioural and psychological symptoms with respect to a wide variety of socio-demographic, cognitive and health-related factors, allowing a detailed investigation of the associations between symptoms and the factors that comprise the definitions of MCI. Our assessment of symptoms has been previously used to report the prevalence and correlates of behavioural and psychological symptoms of dementia in demented and non demented groups, however it is difficult to compare prevalences of symptoms here with those reported using the informant based Neuropsychiatric Inventory (NPI) [15]. Our study was population-based and our estimates were therefore not biased by referral filters. Prevalences were backweighted to account for the study design being representative of the older population of England and Wales without dementia. Symptoms were assessed using interviews conducted with both study participants and their carers, combined with observations made by the interviewer during the course of the interviews. However, neither the severity of symptoms nor the extent to which they were problematic to participants was recorded.

Wandering and confabulation were rare in the non-demented sample and very few individuals lived in institutions making associations between these and other factors difficult to detect. Although the response rate of the study is high at 82%, variables were missing for some subjects. The primary cause of a missing MMSE score in our study is severe cognitive impairment and we excluded individuals with missing MMSE from this investigation. Symptom presence was determined on the basis of any evidence being present from screen or assessment interviews. Where no evidence was present, symptoms were assumed to be absent. Covariate data were missing in only a small proportion of cases and where missing were assumed to be missing at random.

### Depression

Depression has a complex relationship with dementia and cognitive impairment [16]. It is known that depression is a risk factor for dementia [17], but also that depression is a direct cause of cognitive impairment that may not indicate incipient dementia [18]. Depression is further acknowledged as a symptom of dementia with a high incidence in the early stages of dementia [4]. Depression is the most commonly studied non-cognitive correlate of MCI, with estimates of the prevalence of depressive symptoms ranging from 16-40% in population based studies [6]. The varying instruments and threshold for assessment of depression make cross study comparison difficult. The population based Three-City Study found that 16% of 2879 individuals with MCI had some depressive symptoms, 9.2% sub-threshold depression and 2.4% had DSM-IV clinical depression [19]. We found depression to be most common in those with the lowest MMSE scores (17.6%) and more common in MCI (14.5%) than in those with no cognitive impairment (6%) or other cognitive impairments (8.5%).

### Relationship between behavioural and psychological symptoms with cognitive impairment

A subjective memory complaint is the strongest independent predictor of reported symptoms in multivariate analyses. This might be explained by behavioural and psychological symptoms causing memory impairments to be noticed, or a generally high awareness of both cognitive and non-cognitive symptoms. Subjective memory complaints are commonly reported by older people, although previous studies have shown depression to be a stronger predictor of subjective memory complaint than objective cognitive impairments [20]. Subjective memory complaints are required for a clinical diagnosis of MCI, and so those diagnosed with MCI are more likely to report behavioural and psychological symptoms than those who are similarly cognitively impaired in the population.

We found objective memory impairment to be associated with many symptoms in univariate analysis, and some evidence for differing symptom profiles across MCI subtypes, although the power to detect these differences was limited. Hallucinations were significantly more common in A-MCI (7.6%) and M-MCI (8.8%) than N-MCI (2.4%) which contradicts the findings of a clinical study of 120 subjects [21] in which hallucinations were more prevalent in those with N-MCI (6 of 26) than in A-MCI/M-MCI (4 of 94). In the current population-based study the numbers with amnestic and non-amnestic subtypes are more equal with 204 A-MCI/M-MCI and 185 N-MCI which suggests that N-MCI may be under-represented in the clinic and that individuals with N-MCI presenting to clinical services may not be representative and are those with more behavioural and psychological problems.

Lower general cognitive function has been consistently associated with a higher prevalence of behavioural and psychological symptoms in population based studies of mild to moderate dementia [4,22] and in MCI [23]. In the present study most symptoms were more prevalent in those with low MMSE scores. In multivariate analysis the risk of apathy, persecution and misidentification was significantly increased in persons with a lower MMSE score after adjustment for subjective memory complaints and objective memory impairment. The risk of anxiety was associated with a higher MMSE score.

### Correlates of behavioural and psychological symptoms

In univariate analyses, being over 75 years of age had little effect on symptom prevalence. However, in multivariate analyses after adjusting for cognitive function younger participants had a significantly increased risk of depression, feelings of persecution and agitated behaviour, echoing previous findings in dementia [4]. Female subjects were significantly more likely to report depression in common with previous findings [24] while contrary to a previous study of 214 MCI patients we found that men were more likely to report apathy [25], this difference possibly owing to cross study differences in the assessment of apathy.

In those with vascular disease the onset of cognitive impairments may signify early vascular or mixed dementia, with potentially a different profile of behavioural and psychological symptoms. A review of behavioural symptoms in different dementia subtypes found an increase in the risk of depression, emotional labiality, anxiety and apathy in vascular dementia compared to AD, while delusions, delusional misidentification, wandering and restlessness were less frequent in vascular dementia compared with Alzheimer's disease [26]. In our study of the population without dementia the presence of vascular disease significantly increased the risk of depression, apathy, anxiety, irritability and sleep problems, with these associations moderately attenuated after adjusting for cognitive impairment. After adjustment for cognitive impairment agitated behaviour was negatively associated with a history of vascular disease, suggesting an association with non-vascular sources of early cognitive change.

Consistent with previous studies [23,27-29] we observed a positive, significant relationship between ADL impairment and depression, apathy, anxiety, persecution, hallucination, irritability, sleep problems and misidentification. While the first definitions of MCI excluded those with ADL impairments [30], more recent clinical definitions are more inclusive allowing impairment in complex or instrumental ADLs [31] although those with severe ADL impairments may be excluded from a diagnosis.

### Co-occurrence of symptoms

The relationships between some behavioural and psychological symptoms in dementia are well known [32,33], but there are few reports of their co-occurrence in early cognitive impairment [34]. We report a moderate statistically significant association between many pairs of symptoms, which remained after adjusting for cognitive and socio-demographic factors. Factor analysis identified three factors corresponding to misidentification and hallucination; apathy, anxiety and depression; and feelings of persecution and irritability. However, the high uniqueness of each symptom and the significant co-occurrence that was not represented in the factor structure indicates that most of the variance cannot be explained by these factors. In a previous study of the population with dementia in MRC CFAS, similar factors but with stronger patterns of co-occurrence were observed [4].

## Conclusions

Recent reviews have demonstrated large variation in estimates of the prevalence of behavioural and psychological symptoms in MCI [6,7]. This variation can in part be explained by different diagnostic criteria for MCI and use of different study settings. Most symptoms are less prevalent in the population than in clinical samples [6]. We have shown that behavioural and psychological symptoms are related to many of the criteria used in inclusion and exclusion criteria for MCI diagnosis. In particular, those with subjective memory complaints were more likely to report symptoms independently of objective measures of cognitive function and memory impairment. Behavioural or psychological problems may precipitate contact with medical services, which may contribute higher prevalence in tertiary referral centres compared with the general population.

Behavioural and psychological problems are common in dementia [4] and have become accepted as central characteristics of the disorder. Behavioural and psychological symptoms in dementia are at least as problematic for patients and caregivers as cognitive impairments, significantly affecting quality of life and cost of care of people with dementia [1,2]. Furthermore, they currently offer greater opportunities for intervention and management than does cognitive impairment [3]. Current definitions of MCI focus entirely on cognition and may exclude those with psychiatric symptoms on the basis that psychiatric disorders might underlie cognitive impairment which should then not be considered an indicator of incipient Alzheimer's disease. Yet we have shown that many behavioural and psychological symptoms are present in those with mild cognitive impairments with a similar pattern of occurrence to that seen in individuals with dementia. Behavioural and psychological symptoms should be assessed as possible targets for management in cognitively impaired older people. Several studies in patients with MCI have shown that those with behavioural and psychological symptoms have an increased risk of dementia incidence and suggest that non-cognitive symptoms should be a consideration when identifying those in the earliest stages of dementia [6,7]. It remains difficult to differentiate patients with psychological symptoms as a consequence of early dementia from those in whom cognitive impairment is secondary to other psychological conditions. Further population-based longitudinal studies are needed to establish whether behavioural and psychological symptoms can be used alongside memory and other cognitive impairment to improve the identification of those at highest risk of dementia incidence.

## Competing interests

The authors declare that they have no competing interests.

## Authors' contributions

RL, GS and BS designed and conducted the analysis and drafted the manuscript. All authors provided scientific input into the design of the analysis, the interpretation of findings and made scientific revisions to the manuscript.

## Pre-publication history

The pre-publication history for this paper can be accessed here:

http://www.biomedcentral.com/1471-2318/10/87/prepub

## Supplementary Material

Additional file 1 **Table S1**. Univariate associations (odds ratios with 95% confidence intervals) between behavioural and psychological symptoms and socio-demographic, health related and cognitive factors. Click here for file

Additional file 2 **Table S2**. Multivariate associations (odds ratios with 95% confidence intervals, adjusted for all other covariates) between behavioural and psychological symptoms and socio-demographic, health related and cognitive factors. Click here for file

Additional file 3 **Table S3**. Pairwise associations (odds ratios with 95% confidence intervals) between behavioural and psychological symptoms in the population without dementia. Below diagonal are univariate associations, above diagonal are associations adjusted for the presence of all other symptoms, MMSE, age and sex. Statistically significant associations (p < 0.05) are highlighted in bold. Click here for file
